# Problematizing Argumentative Writing in an Iranian EFL Undergraduate Context

**DOI:** 10.3389/fpsyg.2022.862400

**Published:** 2022-06-10

**Authors:** Nasim Ghanbari, Mostafa Salari

**Affiliations:** ^1^Department of English Language and Literature, Persian Gulf University, Bushehr, Iran; ^2^Institute for International Energy Studies, Tehran, Iran

**Keywords:** argumentative writing, academic writing, EFL writing, toulmin argument structure, EAP (English for academic purposes)

## Abstract

Argumentative writing is the most important genre that undergraduate students need to learn to meet their academic requirements. Hence, many studies in different ESL/EFL academic contexts have explored different aspects of argumentative essay at micro text level and also wider educational, contextual and cultural levels. However, majority of these studies have focused on separate aspects of argumentative writing. Therefore, in the absence of studies which examine different variables involved in undergraduate EFL students’ argumentative writing, the present study was conducted by drawing on multiple data sources: students’ perceptions of the argumentative texts, writing teachers’ views on argumentative writing of the students and finally analysis of the structure of the argumentative texts written by the students. For this aim, a total of 66 argumentative essays written by the undergraduate students was analyzed. In addition, a group of 66 undergraduate students majoring in English literature and 20 university writing instructors were interviewed. The findings revealed that the concept of argumentation was poorly conceived and tackled with by the learners. Teachers also counted various grounds that students faced difficulties. Moreover, the structural analysis of the students’ texts showed that they had problems with developing secondary elements of argumentation. In sum, the study discusses that the failure to develop an argumentative essay by the Iranian undergraduate English majors entails several academic, contextual and pedagogical grounds. Implications for improving argumentative writing in the EAP context would be provided.

## Introduction

Academic writing has a recognized significance for both the admission of the students to universities and their academic achievements (Hyland, 2013). Despite the variations in academic writing across the disciplines, argumentative essay is the most important genre in the academic context (Wu, 2006). At the heart of this particular genre lies the ability to develop sound arguments which is an essential skill in the academic context (Toulmin, 1958, 2003; Lea and Street, 1998; Németh and Kormos, 2001; Wolfe, 2011; Wingate, 2012; Rapanta et al., 2013). As Nesi and Gardner (2006) put it, the ability to display critical thinking and develop an argument is commonly pursued across academic texts of different disciplines. However, despite the importance of argumentative essay writing in the academic context, there are many studies in both L1/L2 writing contexts which demonstrate the difficulties the students have with argumentative writing (Qin and Karabacak, 2010; Abdollahzadeh et al., 2017; Altınmakas and Bayyurt, 2019; Saprina et al., 2020; Divsar and Amirsoleimani, 2021; Sundari and Febriyanti, 2021). One of the major struggles the students have is with the concept of argumentation. Many students do not know that they are expected to develop arguments in their essays or they have difficulty with developing an argument in their essays (Davies, 2008; Bacha, 2010; Wingate, 2012). Aside from the particular structure of argumentation which entails a considerable cognitive ability, many studies consider the poor pedagogic activities as an important factor involved in the students’ inability to develop argumentative texts (Wingate, 2012; Abdollahzadeh et al., 2017; Altınmakas and Bayyurt, 2019; Taheri and Nazmi, 2021). Andrews (1995), for example, states that students acquire quite different concepts of argumentation in secondary school. At university, the students are given general guidelines on argumentative writing and they are left to apply them in their argumentative essays. The way argument development is treated in academic context shows the teachers’ uncertainty over the requirements of the argument and at the broader level their tacit knowledge of how to develop an essay in the academic context (Atkinson and Ramanathan, 1995; Lea and Street, 1998; Mitchell and Riddle, 2000; Mutch, 2003; Casanave, 2004; Jacobs, 2005; Wingate, 2012).

The fact is that higher education requires new ways of learning which is qualitatively different from general literacy. This new learning culture which also entails the writing practices of the students demands a kind of writing which is no more a simple language skill for meaning making rather specific academic disciplines require the students to produce text types to meet the requirements of the disciplinary writing. In fact, the ability to argue which is a novel cognitive demand for the majority of the undergraduate students poses difficulties for them since they are still in the process of developing their L2 language proficiency. In addition, EFL/ESL students as people with particular sociocultural and socioeconomic grounds, past schooling histories, established identities, individual learning strategies, etc. need to acculturate to the new academic writing which prominently emphasizes the argumentation and critical thinking (Altınmakas and Bayyurt, 2019; Divsar and Amirsoleimani, 2021). It is clear that the requirements of the new educational context pose a double burden on the undergraduate students as language learners who should also operate with the conventions of the academic discourse.

Much has been written on the linguistic and rhetorical structure of the arguments (Al-Abed-Al-Haq and Ahmed, 1994; Hemmati, 2001; Khiabani and Pourghassemian, 2009; Rashidi and Alimorad Dastkhezr, 2009; Dastjerdi and Samian, 2011; Nimehchisalem et al., 2015). In addition, some other studies (Wingate, 2012; Abdollahzadeh et al., 2017; Altınmakas and Bayyurt, 2019; Saprina et al., 2020; Sundari and Febriyanti, 2021) specifically focused on the students’ problems with developing sound arguments in the higher education contexts. The common thread of all these attempts is the fact that developing argumentative writing as the milestone of academic writing includes a body of linguistic, cultural, pedagogical and contextual factors. However, as passed above, the existing studies in the literature have focused on individual aspects of argumentative writing. This fragmented body of knowledge fails to delineate different linguistic and non-linguistic aspects of argumentative writing. In other words, considering the dynamic and multi-faceted nature of the academic writing, relying on limited data sources would not yield a complete understanding of the argumentative writing of the students. Therefore, the research reported here adopted a wider perspective for studying the argumentative writing of the students by including three main data sources of texts, students and the writing teachers. Putting together the concerns of major stakeholders in academic essay writing, the present study aimed to improve the argumentative writing practices of the students through enacting realistic pedagogical measures. In addition, argumentative essay writing would receive its due attention in the context. The present study was conducted to fulfill these objectives in the context.

## Literature Review

The skill of argumentation has long been considered as one of the basic goals of education (Terenzini et al., 1995; Mitchell and Riddle, 2000). For this, within the past two decades argumentative reading and writing have received growing attention in tertiary education contexts (Feak and Dobson, 1996; Varghese and Abraham, 1998; Helms-Park and Stapleton, 2003; Newell et al., 2011). In fact, as an essential academic skill the students are required to both identify and evaluate the structure of argument and also compose sound arguments. As an evidence, argumentative writing abilities of the students are constantly tested through the recognized International language proficiency tests such as TOEFL, IELTS, GRE (Coffin and Hewings, 2004).

At the heart of argumentative writing lies the concept of argument which has been used differently in the academic discourse. Wingate (2012) states that the concept of argument has been used in three main ways in the scholarly literature. The first view which is based on philosophical syllogism considers argument as individual claims. Here, the argument requires the ability to make inferences out of premises and conclusions (Toulmin, 1958). The second approach defines the development of an argument as developing a position and presentation of it through the logical arrangement of the propositions. Andrews (1995, p. 3) describes this view of argumentation as “a connected series of statements intended to establish a position and implying response to another (or more than one) position.” The last view defines argument as the selecting and evaluating of the content knowledge from the relevant sources to develop the argument (Wu, 2006).

Studies have shown that learners and teachers have vague and partial understanding of the concept of argument. Mitchell et al. (2008), for example, showed that students defined argumentation as a series of “for-and-against” structure put between the introduction and the conclusion sections. Wingate (2012) also found that the students had only partial or incorrect concepts of argument. They were also mostly unaware of the requirements of the argumentative essay, particularly the need to develop their own position in an academic debate. Teachers were also uncertain about the concept of argument as they equaled it with the critical analysis and the expression of opinion. In the study by Lea and Street (1998), academic instructors despite acknowledging the argument as the central element of an essay could not explain the nature of a well-developed argument. In addition, it was found that the instructors had conceptual uncertainties with regard to the nature of argument as they could not differentiate between the argument as individual claims and development of a position. The research has also shown the learners’ difficulties with different components of developing an argument as stated above. The studies have shown that the students could not analyze and evaluate conflicting points of views in the literature (Andrews, 1995); they could not establish a position by making balance between the source and the voice (Groom, 2000) and finally, the learners failed to present their position in a coherent manner, rather they followed a simple formulaic structure to develop their position (Andrews, 1995).

Many studies have also adopted a textual perspective and investigated the learners’ difficulties with the argumentative writing from a textual perspective by analyzing the students’ texts. These studies have shown that English argumentative writing poses rhetorical difficulties for the learners (Al-Abed-Al-Haq and Ahmed, 1994; Hemmati, 2001; Khiabani and Pourghassemian, 2009; Dastjerdi and Samian, 2011; Nimehchisalem et al., 2015; Abdollahzadeh et al., 2017; Saprina et al., 2020). In addition, text-based research that has compared argumentative writing by native and non-native English speakers reveals rhetorical and textual differences, although some similarities have also been found (Choi, 1986, 1988a,b; Lux, 1991; Ferris, 1994; Bouchard, 1996; Kim, 1996; Hinkel, 1999). In addition to the textual perspective, many studies have investigated the processes and strategies that L2 writers use to develop an argumentative essay. Many studies conducted in late 1980s and early 1990s studied the writing processes involved in argumentative writing (Raimes, 1987; Cumming, 1989; Hall, 1990; Whalen and Menard, 1995). Other studies following the same line of inquiry investigated the writing strategies (Leki, 1995; Riazi, 1997; Khaldieh, 2000) the learners used when developing an argumentative essay.

As it can be inferred from the above studies, the students’ problems with argumentative studies have been mostly studied from a textual perspective which focuses on the written products of the students. There are few studies in the literature which have studied the writing difficulties from the writers’ points of view (Zhu, 2009; Wingate, 2012). As an example, Zhu (2009) studied the Mexican graduate students’ argumentative writing difficulties in English and found that the students perceived the most difficult aspect of English argumentative writing to be its rhetorical aspects.

**FIGURE 1:**
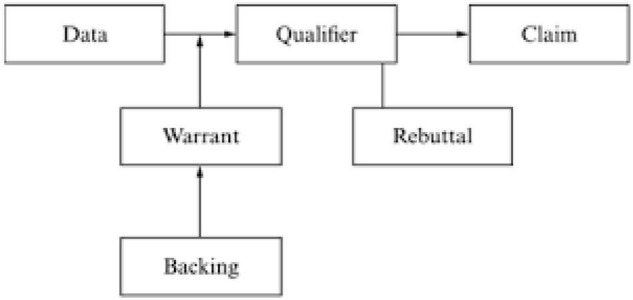
Toulmin’s argument model.

In the last few decades, a number of studies have analyzed the structure of argumentative writing by using Toulmin’s theoretical framework (Toulmin, 1958, 2003; Lea and Street, 1998; Németh and Kormos, 2001; Qin and Karabacak, 2010; Wolfe, 2011; Rapanta et al., 2013; Abdollahzadeh et al., 2017; Sundari and Febriyanti, 2021). The model proposed by British Philosopher Toulmin (1958) has been used to analyze the argumentative writing (Figure 1). According to Toulmin (1958, 2003) every argument is composed of basic components and modifying components. Basic components include claims, data and warrants. Claim considers the conclusion of an argument that should be justified. Data refers to the information used as evidence and warrants which function as a link between claim and data aim to justify the claim using the data. At another level, modifying components include backing, qualifier and rebuttal. The function of backing as the name says is to further support the warrant. The modal terms show the strength of the warrant and rebuttal or counter-claims that the warrant fails to justify the claim based on the data.

The use of Toulmin’s argument components across levels of expertise (Crammond, 1998), the relation of the argument elements and the overall quality of argumentative essays (Qin and Karabacak, 2010; Abdollahzadeh et al., 2017), and the role of goal specification in the frequency of use of argument elements (Page-Voth and Graham, 1999; Ferretti et al., 2000; Nussbaum and Kardash, 2005) have been among the research conducted. As an example, Abdollahzadeh et al. (2017) studying the argumentative behavior of a group of Iranian EFL graduate learners found that the essays were mostly deductively organized. Also, the students used data and claim more frequently compared with the secondary elements of counterarguments and rebuttals. The study further showed that the good surface structure of the arguments does not indicate their soundness. Similarly, Qin and Karabacak (2010) who studied Toulmin’s elements in Chinese EFL argumentative texts, found that data and claim were used significantly more than counterargument and rebuttal.

Along with the above, the literature also shows that argumentative writing in general and argumentation in particular has received little academic support. In fact, despite the importance of argumentation as an important academic achievement for the students in different disciplines (Davies, 2008), the university usually has only relied on few focus courses (if any) and the general feedback of the instructors which do not directly address the concept of argument (Groom, 2000; Altınmakas and Bayyurt, 2019; Divsar and Amirsoleimani, 2021; Taheri and Nazmi, 2021).

Overall, it seems that the research on argumentative writing suffers from the lack of coordinated efforts which consider text, students and teachers in the wider academic contexts. Therefore, adopting a general research perspective would provide pedagogical benefits by making a harmony among teachers’ decisions, students’ needs and the ultimate argumentative text produced. In addition, the findings of this study would prioritize the significance of argumentation as one of the main competencies in the higher education contexts. In fact, the accumulation of evidence on argumentative writing gathered from the major stake holders in the context would help develop sound instructional programs to improve the argumentative ability of the students in the context.

Hence the research reported here aims to extend the current knowledge on EFL argumentative writing by answering the following three research questions:

1. How do Iranian EFL undergraduate students perceive an argumentative essay?
2. What are the common difficulties of Iranian EFL undergraduate students’ argumentative writing identified by writing teachers?
3. What is the structure of argumentative texts written by Iranian EFL undergraduate students?

## Methodology

### Participants

A body of 66 undergraduate students who majored in English language and literature at Persian Gulf University (PGU) in Bushehr, Iran participated in this study. They were selected using the convenience sampling procedure. There were both male and female students in the study (*M* = 15, *F* = 51) and they aged between 20 and 25. At the time of the study, the students had already passed two writing courses of advanced writing and essay writing. All of the participants were native speakers of Persian and had learned English for almost 8 years. The students who come from two intact classes in the context were next given open-ended questionnaire to answer. They were also asked to write argumentative texts for a later structural analysis of the essays.

In addition, a group of 20 experienced university writing instructors participated in this study. The teachers worked as either full-time English professors or visiting instructors at PGU English department. The teachers varied in terms of age, gender and EFL teaching background. There were 13 male and 7 female instructors and they aged between 39 and 56 (*M* = 49) in this study. All the instructors were quite experienced in teaching/assessing writing with a minimum 5 years of teaching and assessing writing experience (Table 1).

**TABLE 1:** Demographics of the participants in the study.

		Categories	*N*	(%)
Students	Gender	Male	15	23
		Female	51	77
	Age	20–22	58	88
		23–25	8	12
	Context of study	PGU	66	100
	Gender	Male	13	65
		Female	7	35
Teachers	Age	39–45	11	55
		46–56	9	45
	Context of teaching	PGU	20	100
	Teaching experience	5–15	8	40
		15–27	12	60
	Context of study	PGU	20	100

### The Study Context

The present study was conducted in the English language and literature department of Persian Gulf University in Bushehr, Iran. Following the English language and literature undergraduate curriculum announced by the Iranian ministry of science, research and technology (MSRT), undergraduate academic writing in this context includes two courses of advanced writing (i.e., paragraph writing) and essay writing. The focus of advanced writing course is to enable the students to learn the essentials of composing simple paragraphs in English. This course is a requirement for the more advanced essay writing course which aims to prepare learners to develop essays in English in different genres including argumentative one (Supplementary Appendix I). English is the medium of instruction and the students are required to handle their assignments in English. In fact, a variety of academic writing types (papers, reviews, summaries, reaction reports, etc.) are needed.

### Instruments

#### Open-Ended Questionnaire

Drawing on the related literature (Lea and Street, 1998; Nesi and Gardner, 2006; Qin and Karabacak, 2010; Wingate, 2012; Nimehchisalem et al., 2015; Abdollahzadeh et al., 2017; Altınmakas and Bayyurt, 2019) and the researcher’s personal experiences with teaching undergraduate writing courses, the researcher developed an open-ended questionnaire in order to elicit the perceptions of the students with regard to argumentative essays (Supplementary Appendix III). The questionnaire included close-response items about the students’ background including demographic information and English essay writing experiences. The next part involved open-ended question items which sought in-depth information on different aspects of argumentative writing. As an example, the students were asked to define argumentative writing and its different parts. After developing the questionnaire, the researcher asked a group of expert colleagues to examine the items for any content or language ambiguities. The researcher applied the experts’ views to modify some of the items. The questionnaire developed in this way were used to collect the students’ views on argumentative writing.

#### Semi-Structured Interviews

A semi-structured interview was developed by the researcher to know how the writing instructors identified the difficulties the learners faced when writing argumentative essays. To develop the interview items, the researcher used the previous studies (Groom, 2000; Zhu, 2009; Wingate, 2012; Altınmakas and Bayyurt, 2019) and also unstructured interviews with some writing instructors in the context. The interview items were prepared in Persian (i.e., native language of the participants) to prevent any probable language hindrances. A panel of experts further examined the questions. After applying the experts’ suggested modifications, a group of 8 questions were selected to be used as interview prompts in this study. The interviews conducted and audio-recorded by the researcher were next transcribed for further analysis.

#### Argumentative Writing Tasks

In order to collect argumentative essays, under an exam-like condition argumentative texts were collected from the students. An argumentative topic was assigned for the students to write. The topic chosen was, “Why did you choose English as your academic major?” To select the topic, a pilot study was done and a group of 30 students were asked to choose among three argumentative topics provided to them. About 70% of the students chose this topic (Why did you choose English as your academic major?). The results of the pilot study showed that many undergraduate students could produce the least required number of paragraphs for an essay (i.e., three). It was assumed that the topic was interesting enough to personally involve the students in developing and reflecting on the topic. Task instruction asked the students to take a position on the topic provided. Overall, a collection of 66 argumentative essays was obtained from the students in the two essay writing groups.

#### Qin and Karabacak’s Rubric

Qin and Karabacak’s (2010) Rubric which was originally based on an adapted Toulmin (2003) model and Nussbaum and Kardash (2005) was employed to identify the argument elements in the students’ essays in this study. In this model, counterargument and rebuttal were divided into two levels of claim and data to provide a more detailed analysis of the argument structure of the arguments. Semantic structure and linguistic elements are used in the rubric to identify different argument elements (Supplementary Appendix II). The rubric has been claimed to reliably identify the argument elements. In this study the inter-rater reliability was 0.89. Also, at the time of disagreement between the raters, data were negotiated until a consensual agreement was obtained.

### Data Collection

**FIGURE 2:**
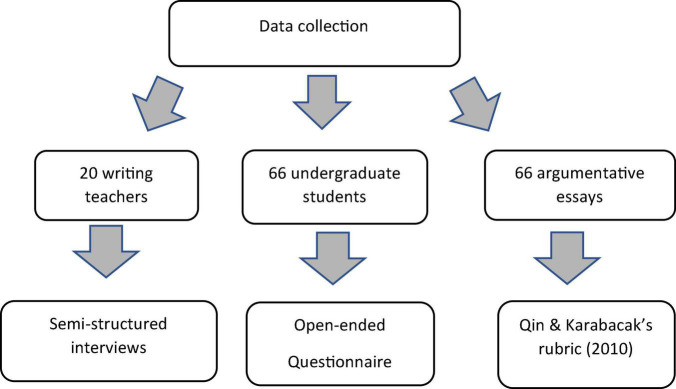
Data collection procedure in the study.

The data for this study was collected during the 2020–2021 academic year. The researcher worked with the students in the two essay writing groups. At the end of the course, the students in both groups were asked to write an argumentative essay as the final exam assignment. Overall, 66 essays were collected. The texts obtained in this way were next analyzed for the argumentative elements based on the Qin and Karabacak’s (2010) rubric. Later, the students were given an open-ended questionnaire which aimed to reveal the students’ perceptions of different aspects of an argumentative essay. The students could answer the items and also provide any further information that they deemed necessary. Finally, a group of 20 writing instructors were asked to participate in a semi-structured interview. Each interview lasted for 60–80 min to give the participants sufficient time to express their views on the difficulties the students encountered with developing an argumentative essay (Figure 2). The interviews obtained in this way were recorded and subscribed for further analysis.

### Data Analysis

Due to multiple data collection procedures used in this study, the researcher used different data analytic measures. The students’ essays were analyzed using Qin and Karabacak’s (2010) rubric. In order to establish the inter-rater reliability, an experienced colleague who was also an experienced writing instructor rated the essays. The measure of inter-rater reliability (0.89) showed high agreement between the two ratings. To analyze the results of the questionnaire and the interviews, the researcher used the qualitative analysis procedure of content analysis. In particular, the conceptual content analysis as the particular content analysis method was used. In conceptual analysis, a concept is chosen for examination and the analysis involves quantifying and counting its presence. Following Dornyei (2007), the data were transcribed verbatim and before coding the researchers carefully read the transcripts several times. Then after several rounds of coding and recoding, the researcher could develop higher-order codes from the data. The major themes obtained in this way were considered as the way the students perceived argumentative writing. In addition, the themes emerged from the analysis of the interviews were considered as the problems the teachers counted the students had with developing argumentative essays. Moreover, the coding of data and forming major themes were next checked with two experts who were familiar with the qualitative content analysis procedure.

## Results

The first research question investigated how the students conceived of an argumentative essay in English. The analysis of results of the open-ended questionnaire provided six major themes. These categories can be seen in Table 2 below based on the frequency of mentions. In fact, a large number of the students did not mention the main characteristics of an argumentative text. As shown below, majority of the students did not know the particular structure of the argumentative essay. They considered the general introduction-body-conclusion as the organization pattern for developing the arguments. The students mentioned that the body of the argument should be developed similar to other essay genres. As an evidence, one of the students described the typical organization of an argument as so,

**TABLE 2:** Students’ perception of the argumentative writing.

Category	Frequency
Argumentative writing is similar to other essay genres.	52
Argumentative writing is for expressing personal opinions.	49
Argumentative writing should persuade the readers.	38
Argumentative writing includes two sides	27
Argumentative writing includes the thesis in the conclusion	25

“*After stating your view in the introduction paragraph, you should explain it in the body paragraph to justify your views.”*

It seems evident that the learners did not know the particular structure of the argumentative essay in English which confirms that they have just generalized their previous writing experience into the argumentative genre.

Moreover, many of the students considered an argumentative text as a kind of writing to express personal opinions. For example, one of the students defined an argumentative essay as so,

*“Argumentative writing means that you should express your views on a controversial topic and also show why you think so.”*

A good of number of the students mentioned that argumentative writing should persuade the readers in the first place. However, their responses did not show that they have the argumentative structure in mind. As evidence, only a few of the students considered argument to include multiple views, rather their responses showed that by argumentative text, they aimed a persuasive explanatory essay in which they should provide sufficient grounds to convince the readers of their claims posed early in the text. This structure, in the respondents’ views, was expressed in a first-person writer-only text.

In addition, some of the students stated that there are always two parties involved in argumentative writing. However, as mentioned before, they were unable to explain how such a dialog was represented in the argumentative text. In fact, the inclusion of two sides in an argument shows that the students fail to consider multiple resources in developing an argument. It can be inferred that they did not know that evaluating and analyzing the resources is indispensable in developing a robust argument in English. Further, the second party which was an imaginary figure developed by the writer was restrained to pose particular views which were part of the writer’s imagination.

It was in this imaginary dialog that the students believed that they should persuade the readers to accept their personal views. In fact, the students adopted an empty notion of the persuasion. The following excerpt by one of the students clearly shows the way the students conceived the persuasion in argumentative writing:

“*In the body I should state ideas that would fortify my claim posed earlier. I believe this would have two functions. The first is that I have supported my claim and the second is to justify those who disagree.”*

Relying on their L1 background, some students considered a different structure for the argumentative text in which the thesis statement appeared in the conclusion section of the essay. This assumption was interesting since the students know the structure of English essays in which the thesis statement appeared in the introduction paragraph but when it came to argumentative writing, they believed that the claim as the product of argumentation should appear after enough supports have been posed. All in all, the way the students perceived the argumentative essay revealed that the students had a partial and narrow concept of the argumentative writing.

As mentioned, teachers also participated in semi-structured interviews which aimed to identify the kind of difficulties the students faced in argumentative writing. The teachers’ comments were grouped into six categories as shown in Table 3 below. The most frequently mentioned problem by the teachers was lack of structure. The teachers believed that the students failed to organize their ideas into a logical structure. The teachers stated that in many cases the texts were jumpy and could not present the students’ ideas in a coherent way. The teachers’ comments further showed that the problem with structure related to the students’ inability to state their position (i.e., claim) in a sound argumentative structure. The next most frequent problem mentioned by the teachers was the lack of evidence in the students’ argumentative texts. The teachers claimed that the students failed to use sources to support their claims. They used their own personal opinions or popular, wise sayings instead. Similarly, the students could not evaluate the information to write the relevant information in their text. In fact, the teachers stated that the students mislead their line of argumentation by providing lots of information which usually do not fit their text. In other words, students who aim to improve their arguments by displaying the range of their knowledge usually provide lots of irrelevant information which does not help the argument.

**TABLE 3:** Teachers’ views on students’ problems with argumentative writing.

Lack of structure
Lack of evidence (unable to use/evaluate the related sources)
Lack of critical analysis
Problems with basic writing skills (sentence construction skills, paragraph development skills, etc.)
Poor L1 & L2 pre-university writing experience
Marginal role of argumentative writing experience/practice in the context

The next problem which was not unrelated to the previous problem was the students’ inability to critically analyze the information. Critical analysis of data which is at the core of argumentation ability concern the students’ ability to provide opposing views (i.e., counterarguments) and be able to refute them (i.e., rebuttal). In the words of teachers when the students could not provide relevant evidence, then they could not critically evaluate the information and hence develop integrative and coherent arguments. One of the teachers who viewed these two problems as related stated as so,

*“The most challenging and troublesome flaw with learners’ argumentative essays stems from their lack of critical thinking abilities. My 14-year experience in academic writing convinces me to uphold that what hampers the process of argumentative writing is the students’ inability to gather data, think independently, and establish a stance through logical development of ideas.”*

Moreover, a good number of the teachers claimed that the students suffered from basic literacy skills. In particular, they referred to poor sentence construction skills in English. They believed that this basic deficiency negatively affected the students’ ability to develop any written pieces including argumentative essays in English. The teachers emphasized that English program in the Iranian EFL context should further focus on improving the basic language skills such as sentence construction skills, paragraph development skills which would affect subsequent writing courses in the program. Two of the teachers described part of this problem as so,

*“Most of the students have not yet mastered sentence construction skills. They have problems making grammatical sentences. Moreover, they are unable to develop a topic while maintaining unity and coherence.”*

*“Some of these problems concern the students’ lack of ability to write good sentences, choose proper vocabulary, use suitable cohesive devices, not to mention the mechanics of writing such as punctuation, capitalization, and spelling. These can adversely affect any kind of writing, especially argumentative essays.”*

The other difficulty concerned L1 and L2 pre-university writing experience. In teachers’ ideas, the students had only a blurred picture of Persian (L1) idea development in writing in which the conclusion received the most importance. The students did not know how to develop a well-formed argument which caused them to develop explanatory essays instead of argumentative one most of the time. One of the teachers who particularly emphasized the poor argumentative writing tradition in the students’ L1 referred to the problem as so,

*“This mostly has its roots in the students’ not being trained in their pre-university years to question the phenomena, think critically, welcome opposition, analyze, and think independently regardless of sources, disagreeing voices, and conventional norms and values.”*

In teachers’ ideas, Iranian EFL students experienced their first serious writing practices in English at university. It is evident that argumentative writing which relies on solid development principles would be particularly unfamiliar and challenging for the students. The teachers also mentioned that Iranian pre-university English language instruction followed a traditional grammar-based methodology in which reading comprehension skills received the most emphasis. Despite the national change into foreign language curriculum which emphasized a communicative approach to language teaching, the reality of practice underestimated the writing courses.

The last problem identified by the teachers was that argumentative writing despite its importance in the academic context did not receive its due priority both in the national curriculum and by the writing teachers. Argumentative writing was treated simply as a genre similar to other essay types. Consequently, it was not the focus of explicit instruction. Therefore, the students developed a partial and fuzzy concept of argumentative writing. As evidence, some teachers referred to poor pedagogical practices of the instructors in the composition courses which could add to the problem. One of the teachers described the problem as so,

*“We, as teachers, might not have taken the writing courses seriously. It may be due to the fact that we have our own problems outside the classroom, in our private lives. Enhancing writing skill requires a long process. On the one hand the student must strive and practice to learn. This is something which does not happen with our students. On the other hand, we, teachers, must put more time to correct and comment on the students’ writing. Of course, one reason, or justification can be the crowded writing classes. It would be a burdensome task for the teacher to put a lot of time on the students’ writing.”*

Some other teachers even referred to teachers’ incorrect concepts of argumentation. In their ideas, many EFL teachers have vague ideas of argumentation which shows a broader unawareness of the requirements of argumentative essay in English among them. For example, one of the teachers referred to her personal experience to show that many EFL writing instructors themselves have a tacit knowledge of the rhetorical requirements of the argumentative essays:

*“I have problems with argumentation in my writing let alone the students. Personally, I think one of the reasons that my articles as a member of the Iranian EFL writers are rejected is that we are not aware of the rhetoric of the English language. For example, we have problems in presenting our arguments in a coherent way. We either take many points for granted in writing or provide too much information which includes lots of irrelevant data.”*

Last, in order to analyze the structure of the students’ argumentative essays, two types of analyses were conducted. First, rhetorical organization of the essays were examined. This was done to find out if the students developed any position (i.e., claim) in their essays and if so, what were the rhetorical patterns preferred by them. Next, elements of the students’ arguments including claim, data, counterclaim, counterclaim data, rebuttal and rebuttal data were examined based on Qin and Karabacak’s (2010) rubric. In this rubric semantic structure and linguistic elements are used to identify the argumentative elements. For example, phrases such as in my opinion, I think, I believe refers to claim. Also, counterarguments and rebuttals are identified through phrases such as, however, while…although; in spite of the fact that….

**FIGURE 3:**
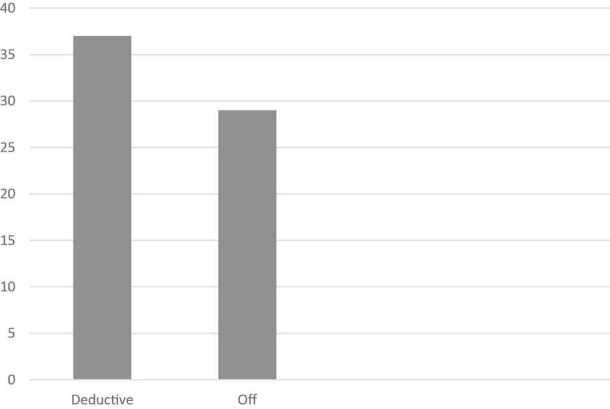
Rhetorical organization types in students’ argumentative essays in this study.

The analysis of the texts showed that almost half of the students (56%, *n* = 37) stated their position clearly at the beginning of their essays. However, 43% (*n* = 29) of the students had not adopted an explicit stance on the topic. In sum, the overall rhetorical organization of the essays was as deductive (56%) and off (43%) (Figure 3).

In the next stage of analysis following Qin and Karabacak’s (2010) (Supplementary Appendix I), different elements of the arguments such as claim, data counterargument claim, counterargument data, rebuttal claim and rebuttal data were identified and their associated frequencies were calculated. The analysis of the texts showed that although more than half of the students (56%) could take a stance in their essays, still a good number of them failed to develop an explicit position in their essays. According to Toulmin (1958), a claim should be supported by its relevant data in order to be considered as a claim; otherwise it is just a piece of personal opinion. The analysis of the students’ texts showed that nearly half of the students’ essays included elements of data. As Table 4 below shows although there was a salient difference in the frequency of use of claim and data as the basic argument elements and the counterarguments and rebuttals as the secondary argument elements, the undergraduate students had difficulties for developing sound claims and their supporting data. The greater number of claims compared with the data confirmed the students’ inability to develop sound claims further supported by the relevant data.

**TABLE 4:** Frequency of argument elements in the students’ argumentative essays.

Components	*N*	(%)
Claim	37	56
Data	27	41
Counterargument claim	13	20
Counterargument data	11	17
Rebuttal claim	4	6
Rebuttal data	1	1.5

## Discussion

The present study investigated argumentative writing in the Iranian undergraduate EFL context. Adopting a holistic perspective, the study examined the issue through exploring three data sources: students, writing instructors and argumentative texts. The findings obtained from the students’ perceptions of the argumentative writing, the teachers’ views on the students’ problems in the argumentative writing and finally the structural analysis of the students’ argumentative texts revealed that the Iranian EFL students suffer from considerable problems in different areas of argumentative writing. The analysis of the data gathered through three different sources showed considerable convergence which implies that EFL academic writing faces similar challenges. A closer look at the findings revealed that the students have difficulty with argumentation in the first place. They conceive of arguments as individual claims. However, they did not know how to put their ideas into a coherent, logical structure. The analysis of the students’ argumentative essays and also what the instructors counted as main difficulties showed that the students failed to meet the basic requirements of developing an argumentative text such as gathering enough evidence from different opposing sources, evaluate and critically analyze them and next present them through a sound organization.

This observation can be explained on several grounds. First and the most important one is that Iranian EFL students live in a literacy culture in which written mode has a marginal role. A deeper look at the pre-university L1 and L2 writing instruction reveals that the Iranian education system provides vague and limited writing instruction which does not offer any contribution for the students’ educational outcomes. In a context where writing does not bear any role in the educational fulfillment of the students, the students would develop their particular writing styles to meet the least writing they are expected to do. Argumentative writing requires a robust reasoning tradition in place before practicing with this genre. It goes without saying that the Iranian EFL students with vague conceptions of argumentative writing and poor reasoning abilities would encounter several difficulties when developing the argumentative texts. Several studies (Kobayashi and Rinnert, 2002; Altınmakas and Bayyurt, 2019; Taheri and Nazmi, 2021) reported similar L1 writing situation. For example, Altinmakas and Bayyurt (2019) claimed that the Turkish pre-university education system does not provide the students with the needed writing knowledge and skills which the students can later use in the academic context. Similarly, Kobayashi and Rinnert (2002) showed that despite the importance attached to L1 writing instruction in Japanese education system, the actual classroom practice put less emphasis on the writing skill compared with the reading skill mainly due to the impact of university entrance exam. Taheri and Nazmi (2021) also showed how providing scaffolding strategies while writing instruction would affect the argumentative writing ability of the participants in terms of the total organization and linguistic accuracy. Therefore, the marginal role of L1 writing among other skills would exert its negative impact on the academic writing at university.

On a similar ground, it is said that the students should take responsibility for their learning in the tertiary level education. This is a difficult task for the students who have experienced teacher-fronted, exam-oriented education system before entering the university. The past learning habits of the students only emphasized rote learning in a passive way and little intellectual engagement was required. It is clear that the students encounter serious problems when they should both critically reflect on the content and manage the form of the language when developing argumentative essays.

The students’ perception of the argumentative writing in this study showed that the students had a partial understanding of the argumentative genre. Since Iranian EFL students receive their first academic writing instruction in the university, it can be claimed that writing teachers are also involved. The analysis of the students’ argumentative texts partly reflects the way they have been instructed. From these observations it can be inferred that the L2 writing teachers mainly focus on the students’ immediate L2 writing needs such as grammatical knowledge and mechanics of writing which reflects the teachers’ lack of competence in developing argumentative essays. Poor qualifications of EAP practitioners has been a recurrent theme in many studies (Belcher, 2013; Basturkmen, 2019). Despite the global increase of EAP courses, there is a paucity of research on EAP practitioners’ knowledge base (Kaivanpanah et al., 2021). In a context where the teachers receive no explicit training, they are left alone with the complex task of teaching EAP. Particularly when it comes to teaching writing, the same confusion persists. Needless to say, academic writing as a multi-layered difficult task is simply treated on impressionistic and subjective grounds (Wingate, 2012). In the absence of professional development programs, teachers proceed by drawing on their partial knowledge of the EAP writing.

According to Hyland (2011) learning to write involves five kinds of knowledge: content knowledge, system knowledge, process knowledge, genre knowledge and context knowledge. It goes without saying that EFL student writers with a poor L1 writing history cannot achieve this set of knowledge to develop conceptual, interpretive skills. According to Currie (1993), the writing process is a socialization process in which the writers develop different writing skills and conventions throughout the time. The undergraduate students in this study who have recently received the academic writing instruction seem to have a long way to learn the critical skills needed in developing sound arguments. Therefore, EAP writing teachers should develop a more tolerant attitude to the students’ difficulties with an argumentative text. The teachers should remember that academic discourse culminates in the social, cognitive and epistemological context of particular disciplinary contexts (Hyland, 2009). Making judgments about the students’ argumentative writing as the most important academic text genre in a context where the students face the first rule-governed and serious writing practices does not provide a comprehensive picture of their academic writing practices. Therefore, teachers in similar contexts to the present study should consider academic writing as part of a complex system which progresses in time.

The next ground which is not unrelated to above is the poor general language ability of the EFL undergraduate learners in this study. In fact, the students who still have problems with the basics of sentence construction in English would be unable to synthesize larger chunks of the language into a coherent, logical whole. In other words, English academic writing program should put more weight on improving the basic writing skills of the students such as sentence development and paragraph development along with emphasizing more advanced academic writing conventions.

## Conclusion

This study aimed to provide a general picture of EFL argumentative writing as the most important kind of academic writing. The data elicited from three different sources revealed that argumentative writing is poorly treated in the context. Findings of the present study can have several implications. At the most basic level, the study calls for a change in the educational value attached to the academic writing in general and argumentative writing in particular. At the wider level, curriculum developers should put more emphasis on pre-university L1 and L2 writing instruction programs. This change should make the writing meaningful for the students. Teachers who implement the curricular goals also should involve students in writing tasks in a way that they can think, and critically evaluate the originality and relevance of the ideas for further development of sound arguments. This systematic integration of L1 and L2 writing from the early years of writing instruction can considerably improve the writing practices of the students in the following tertiary level education as well.

Moreover, findings of the study would clarify the way argumentation in English is perceived by the students. This will aid the writing instructors to provide more focused instructional programs for the students. As the findings showed, the sound organization of the essay which was mainly concerned with the development of the arguments was the recurring problem mentioned by different parties who took part in this study. To fix this fundamental problem is beyond the mechanical and the surface linguistic aspects, rather the students should learn how to think, critically evaluate the sources and organize different parts of an argument. Such abstract conceptual activities require a writing program that confirms the primacy of the written discourse in the academic context first and then design writing activities to improve the argumentative writing of the students in the long run. Teacher professional development programs have a critical role in preparing the pre-service teachers to fulfill the above task. Prospective EAP writing teachers should also know that academic writing is not a separate activity that can be worked on individually, rather, it is a dynamic and evolving skill that should be taught by drawing on several grounds.

Present study also suffers from a number of limitations. The study was conducted in a single university in Iran which narrows the scope of the study. Further studies should be designed to include more universities in the context. Similarly, the study was conducted with a limited sample of students, teachers and texts which restrict the generalizability of the findings.

Findings of the present study also provide new directions for future research projects. For example, comparing the results of the present study with ESL and ENL contexts would reveal to what extent the context may affect the academic writing. In sum, the study would also call for more studies on the EFL academic writing in general and argumentative writing in particular. It is expected that this focus would improve the academic achievement of the students in the academic settings where the written mode prevails.

## Data Availability Statement

The raw data supporting the conclusions of this article will be made available by the authors upon the request.

## Ethics Statement

Ethical review and approval was not required for the study on human participants in accordance with the Local Legislation and Institutional Requirements. The patients/participants provided their written informed consent to participate in this study.

## Author Contributions

Both authors listed have made a substantial, direct, and intellectual contribution to the work, and approved it for publication.

## Conflict of Interest

The authors declare that the research was conducted in the absence of any commercial or financial relationships that could be construed as a potential conflict of interest.

## Publisher’s Note

All claims expressed in this article are solely those of the authors and do not necessarily represent those of their affiliated organizations, or those of the publisher, the editors and the reviewers. Any product that may be evaluated in this article, or claim that may be made by its manufacturer, is not guaranteed or endorsed by the publisher.

## References

[B1] Abdollahzadeh E. Amini Farsani M. Beikmohammadi M. (2017). Argumentative writing behavior of graduate EFL learners. *Argumentation* 31 641–661.

[B2] Al-Abed-Al-Haq F. Ahmed A. S. E. A. (1994). Discourse problems in argumentative writing. *World Englishes* 13 307–323. 10.1111/j.1467-971X.1994.tb00318.x

[B3] Altınmakas D. Bayyurt Y. (2019). An exploratory study on factors influencing undergraduate students’ academic writing practices in Turkey. *J. Engl. Acad. Purp.* 37 88–103.

[B4] Andrews R. (1995). *Teaching and Learning Argument.* London: Cassell.

[B5] Atkinson D. Ramanathan V. (1995). Cultures of writing: an ethnographic comparison of L1 and L2 university. *TESOL Q.* 29 539–568.

[B6] Bacha N. (2010). Teaching the academic argument in a university EFL environment. *J. English Acad. Purp.* 9 229–241. 10.1016/j.jeap.2010.05.001

[B7] Basturkmen H. (2019). ESP teacher education needs. *Lang. Teach.* 52 318–330. 10.1017/S0261444817000398

[B8] Belcher D. (2013). “The future of ESP research: resources and access and choice,” in *Handbook of English for Specific Purposes*, eds Paltridge B. Starfield S. (Boston, MA: Blackwell), 535–552. 10.1002/9781118339855.ch28

[B9] Bouchard R. (1996). Argumentative competence and written production in foreign and native languages. *Lang. Fr.* 112 88–105. 10.3406/lfr.1996.5362

[B10] Casanave C. P. (2004). *Controversies in Second Language Writing: Dilemmas and Decisions in Research and Instruction.* Ann Arbor, MI: The University of Michigan Press.

[B11] Choi Y. H. (1986). A study of coherence in Korean speakers’ argumentative writing in English. *Stud. Linguist. Sci.* 16 67–94.

[B12] Choi Y. H. (1988a). Text structure of Korean speakers’ argumentative essays in English. *World Englishes* 7 129–142. 10.1111/j.1467-971X.1988.tb00226.x

[B13] Choi Y. H. (1988b). Textual coherence in english and Korean: an analysis of argumentative writing by American and Korean students. *Diss. Abstr. Int.* 50:429.

[B14] Crammond J. (1998). The uses and complexity of argument structures in expert and student persuasive writing. *Writt. Commun.* 15 230–268.

[B15] Coffin C. Hewings A. (2004). “IELTS as preparation for tertiary writing: distinctive interpersonal and textual strategies,” in *Analyzing Academic Writing: Contextualized Frameworks*, eds Ravelli L. J. Ellis R. A. (London: Continuum), 153–171.

[B16] Cumming A. (1989). Writing expertise and second language proficiency. *Lang. Learn.* 39 81–141. 10.1111/j.1467-1770.1989.tb00592.x

[B17] Currie P. (1993). Entering disciplinary community: conceptual activities required to write for one introductory university course. *J. Second Lang. Writ.* 2 101–117.

[B18] Dastjerdi H. V. Samian S. H. (2011). Quality of Iranian EFL learners’ argumentative essays: cohesive devices in focus. *Mediterr. J. Soc. Sci.* 2:65.

[B19] Davies W. M. (2008). ‘Not quite right’: helping students to make better arguments. *Teach. High. Educ.* 13 327–340. 10.1080/13562510802045352

[B20] Divsar H. Amirsoleimani K. H. (2021). Developing voice in EFL learners’ argumentative writing through dialogical thinking: a promising combination. *J. Lang. Horiz.* 4 145–166.

[B21] Dornyei Z. (2007). *Research Methods in Applied Linguistics.* Oxford: Oxford University Press.

[B22] Feak C. Dobson B. (1996). Building on the impromptu: a source-based academic writing assessment. *College ESL* 6 73–84.

[B23] Ferretti R. P. MacArthur C. A. Dowdy N. S. (2000). The effects of an elaborated goal on the persuasive writing of students with learning disabilities and their normal achieving peers. *J. Educ. Psychol.* 92 694–702.

[B24] Ferris D. (1994). Rhetorical strategies in student persuasive writing: differences between native and non-native English speakers. *Res. Teach. English* 28 45–65.

[B25] Groom N. (2000). “A workable balance: self and source in argumentative writing,” in *Learning to Argue in Higher Education*, eds Mitchell S. Andrews R. (Portsmouth: Boynton/Cook Heinemann), 65–145.

[B26] Hall C. (1990). Managing the complexity of revising across languages. *TESOL Q.* 24 43–60. 10.2307/3586851

[B27] Helms-Park R. Stapleton P. (2003). Questioning the importance of individualized voice in undergraduate L2 argumentative writing: an empirical study with pedagogical implications. *J. Second Lang. Writ.* 12 245–265.

[B28] Hemmati F. (2001). *Vocabulary Problems in the EFL Writing of Iranian Students: Taxonomies and strategies Doctoral Dissertation.* Colchester: University of Essex.

[B29] Hinkel E. (1999). “Objectivity and credibility in L1 and L2 academic writing,” in *Culture in Second Language Teaching and Learning*, ed. Hinkel E. (Cambridge: Cambridge University Press), 90–108.

[B30] Hyland K. (2009). *Academic Discourse: English in a Global Context.* London: Continuum International Publishing Group.

[B31] Hyland K. (2011). “Learning to write: issues in theory, research, and pedagogy,” in *Learning-to-Write and Writing-to-Learn in an Additional Language*, ed. Manchon R. M. (Amsterdam: John Benjamins Publishing), 17–35.

[B32] Hyland K. (2013). Writing in the university: education, knowledge and reputation. *Lang. Teach.* 46 53–70.

[B33] Jacobs C. (2005). On being an insider on the outside: new spaces for integrating academic literacies. *Teach. High. Educ.* 10 475–487. 10.1080/13562510500239091

[B34] Kaivanpanah S. Alavi S. M. Bruce I. (2021). EAP in the expanding circle: exploring the knowledge base, practices and challenges of Iranian EAP practitioners. *J. English Acad. Purp.* 50:100971. 10.1016/j.jeap.2021.100971

[B35] Khaldieh S. (2000). Learning strategies and writing processes of proficient vs. less-proficient learners of Arabic. *Foreign Lang. Ann.* 33 522–534. 10.1111/j.1944-9720.2000.tb01996.x

[B36] Khiabani M. N. Pourghassemian H. (2009). Transfer of l1 organizational patterns in argumentative writings of Iranian EFL students: implications for contrastive rhetoric. *J. Teach. English Foreign Lang. Lit.* 1 23–38.

[B37] Kim J. W. (1996). Linguistics, rhetorical, and strategic aspects of Korean students’ persuasive writing in English. *Diss. Abstr. Int.* 57:609.

[B38] Kobayashi H. Rinnert C. (2002). High school student perceptions of first language literacy instruction: implications for second language writing. *J. Second Lang. Writ.* 11 91–116.

[B39] Lea M. Street B. (1998). Student writing in higher education: an academic literacies approach. *Stud. High. Educ.* 23 157–172. 10.1080/03075079812331380364

[B40] Leki I. (1995). Coping strategies of ESL students in writing tasks across the curriculum. *TESOL Q.* 29 235–260. 10.2307/3587624

[B41] Lux P. (1991). Discourse styles of Anglo and Latin American college students’ writers. *Diss. Abstr. Int.* 52:2128A.

[B42] Mitchell S. Riddle M. (2000). *Improving the Quality of Argument in Higher Education: Final Report. School of Lifelong Learning and Education.* London: Middlesex University.

[B43] Mitchell S. Prior P. Bilbro R. Peake K. See B. H. Andrews R. (2008). A reflexive approach to interview data in an investigation of argument. *Int. J. Res. Method Educ.* 31 229–241. 10.1080/17437270802416806

[B44] Mutch A. (2003). Exploring the practice of feedback to students. *Act. Learn. High. Educ.* 4 24–38. 10.1177/1469787403004001003

[B45] Németh N. Kormos J. (2001). Pragmatic aspects of task-performance: the case of argumentation. *Lang. Teach. Res.* 5 213–240.

[B46] Nesi H. Gardner S. (2006). “Variation in disciplinary culture: university tutors’ views on assessed writing tasks,” in *Language, Culture and Identity in Applied Linguistics*, eds Kiely R. Rea-Dickins P. Woodfield H. Clibbon G. (London: BAAL/Equinox), 99–117.

[B47] Newell G. E. Beach R. Smith J. VanderHeide J. (2011). Teaching and learning argumentative reading and writing: a review of research. *Read. Res. Q.* 46 273–304.

[B48] Nimehchisalem V. Abbasi M. M. Ebrahimzadeh A. Rezvani Kalajahi S. A. (2015). Iranian English as a Foreign Language (EFL) learners’ argumentative writing performance in private language institutes. *Asian Soc. Sci. J.* 11 96–103. 10.5539/ass.v11n15p96

[B49] Nussbaum E. M. Kardash C. A. M. (2005). The effects of goal instructions and text on the generation of counterarguments during writing. *J. Educ. Psychol.* 97, 157–169.

[B50] Page-Voth V. Graham S. (1999). Effects of goal setting and strategy use on the writing performance and self-efficacy of students with writing and learning problems. *J. Educ. Psychol.* 91 230–240.

[B51] Qin J. Karabacak E. (2010). The analysis of Toulmin elements in Chinese EFL university argumentative writing. *System* 38 444–456.

[B52] Raimes A. (1987). Language proficiency, writing ability, and composing strategies: a study of ESL college student writers. *Lang. Lear.* 37 439–467. 10.1111/j.1467-1770.1987.tb00579.x

[B53] Rapanta C. Garcia-Mila M. Gilabert S. (2013). What is meant by argumentative competence? An integrative review of methods of analysis and assessment in education. *Rev. Educ. Res.* 83 483–520. 10.3102/0034654313487606

[B54] Rashidi N. Alimorad Dastkhezr Z. (2009). A comparison of English and Persian organizational patterns in the argumentative writing of Iranian EFL students. *J. Linguist. Intercult. Educ.* 2 131–52. 10.29302/jolie.2009.2.1.9

[B55] Riazi A. (1997). Acquiring disciplinary literacy: A social-cognitive analysis of text production and learning among Iranian graduate students of education. *J. Second Lang. Writ.* 6 105–137. 10.1016/S1060-3743(97)90030-8

[B56] Saprina C. M. Rosyid A. Suryanti Y. (2020). Difficulties in developing idea encountered by students in writing argumentative essay. *J. English Lang. Stud.* 5 1–7. 10.30998/scope.v5i2.8544

[B57] Sundari H. Febriyanti R. H. (2021). The analysis of Indonesian EFL argumentative writing using Toulmin’s model: The structure and struggles from the learners. *J. English Lang. Teach.* 5 67–78.

[B58] Taheri P. Nazmi R. (2021). Improving EFL learners’ argumentative writing ability: teacher vs. peer scaffolding. *Teach. English Lang.* 15 299–333.

[B59] Terenzini P. T. Springer L. Pascarella E. T. (1995). Influences affecting the development of students’ critical thinking skills. *Res. High. Educ.* 36 23–39. 10.1007/BF02207765

[B60] Toulmin S. (2003). *The Uses of Argument*, Vol. 2. Cambridge: Cambridge University Press.

[B61] Toulmin S. (1958). *The Uses of Argument.* Cambridge: Cambridge University Press.

[B62] Varghese S. A. Abraham S. A. (1998). Undergraduates arguing a case. *J. Second Lang. Writ.* 7 287–306.

[B63] Whalen K. Menard N. (1995). L1 and L2 writers’ strategic and linguistic knowledge: a model of multiple-level discourse processing. *Lang. Learn.* 45 381–418. 10.1111/j.1467-1770.1995.tb00447.x

[B64] Wingate U. (2012). ‘Argument!’ Helping students understand what essay writing is about. *J. English Acad. Purp.* 11 145–154. 10.1016/j.jeap.2011.11.001

[B65] Wolfe C. R. (2011). Argumentation across the curriculum. *Writ. Commun.* 28 193–219.

[B66] Wu S. M. (2006). Creating a contrastive rhetorical stance: investigating the strategy of problematization in students’ argumentation. *RELC J.* 37 329–353. 10.1177/0033688206071316

[B67] Zhu W. (2009). Performing argumentative writing in English: difficulties, processes, and strategies. *TESL Can J.* 19 35–50. 10.3390/antibiotics9030115 32156082 PMC7148451

