# Supplementary material for: Problematizing Argumentative Writing in an Iranian EFL Undergraduate Context
Source: Front Psychol. 2022 Jun 10;13:862400. doi: 10.3389/fpsyg.2022.862400 (PMC9226766; doi:10.3389/fpsyg.2022.862400)
Supplement: Supplementary file 1 [file Data_Sheet_1.docx]

**Appendix I.** Course description for the two undergraduate writing courses in the Iranian EFL undergraduate program

| ***Advanced Writing*** | ***Essay Writing*** |
| --- | --- |
| **Type of the course**: Compulsory  **No. of credits**: 2 | **Type of the course**: Compulsory  **No. of credits**: 2 |
| **Course Objectives**     - To familiarize the students with principles, basics and features of paragraph writing in English - Students be able to identify and write different kinds of paragraphs in English | **Course Objectives**   - Teaching to write essays in different genres - Familiarity with different stages of writing (e.g. pre-writing, drafting, revising, editing) - Critical analysis of well-known essays |
| **Main areas to be covered**   - Paragraph structure - Organization and coherence patterns - Cohesive devices - Different kinds of supports (e.g. anecdotes, examples, details, etc.) - Punctuation rules - Process paragraphs - Compare & contrast paragraphs - Summary paragraphs | **Main areas to be covered**   - A review of paragraph writing - Collecting data - Planning - Structure of an English essay - Essay writing procedure - How to write body of the essay - How to write conclusion - Writing essays in different genres (e.g. explanatory, Compare & Contrast, persuasive, literary essays) |

**Appendix II.** Definitions and examples of six Toulmin elements taken from Qin and Karbacak’s (2010)


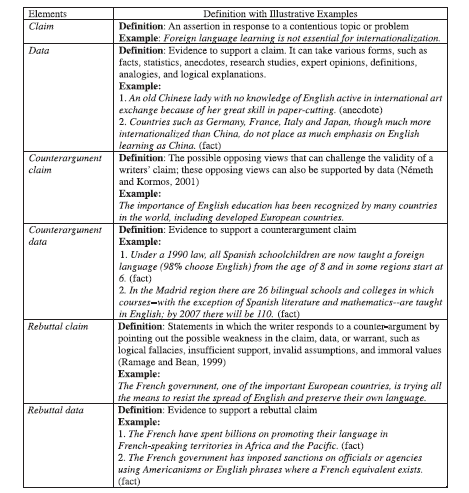


**Appendix III. Argumentative writing questionnaire**

**Dear respondent,**

As part of a research study, the following items aim to find out how you perceive argumentative writing. Please provide your answers in the spaces provided. Your information would be quite confidential and would be used only for research purposes.

Name (optional):

Age: Major:

Gender: Male □ Female □

Academic writing courses passed so far:

Any other writing courses:

1. In your idea, what is argumentative writing?

2. Does argumentative writing differ from other types of writing? If yes, in what ways?

3. What is a typical structure for an English argumentative essay?

4. How do you develop different parts of an argumentative essay?

5. What is the aim of argumentative writing?

6. How do you consider argumentative writing in the academic setting?
